# Statin prevents chondrocyte aging and degeneration of articular cartilage in osteoarthritis (OA)

**DOI:** 10.18632/aging.100213

**Published:** 2010-10-25

**Authors:** Kazuo Yudoh, Rie Karasawa

**Affiliations:** Department of Frontier Medicine, Institute of Medical Science, St. Marianna University School of Medicine, Kawasaki, Japan

**Keywords:** osteoarthritis, chondrocyte, statin, cellular aging, articular cartilage

## Abstract

Recent reports have shown that statin (HMG-CoA reductase inhibitors) may have the potential to inhibit inflammatory arthritis. More recently, the idea that chondrocyte aging is closely associated with the progression of cartilage degeneration has been promulgated. Here, we demonstrate the potential of statin as protective agents against chondrocyte aging and degeneration of articular cartilage during the progression of osteoarthritis (OA), both in vitro and in vivo. The OA-related catabolic factor, IL-1β induced marked downregulation of cellular activity, expression of a senescent biomarker, specific senescence-associated β-galactosidase activity and shortening of the cellular lifespan in chondrocytes. In contrast, treatment with statin inhibited the IL-1β-induced production of cartilage matrix degrading enzymes (metalloprotease-1 and -13) and cellular senescence in of chondrocytes in vitro. In addition, this statin accelerated the production of cartilage matrix proteoglycan in chondrocytes. The in vivo study was performed on the STR/OrtCrlj mouse, an experimental model which spontaneously develops an osteoarthritic process. In this mouse model, treatment with statin significantly reduced the degeneration of articular cartilage, while the control knee joints showed progressive cartilage degeneration over time. These findings suggest that statin may have the potential to prevent the catabolic stress-induced chondrocyte disability and aging observed in articular cartilage. Our results indicate that statin are potential therapeutic agents for protection of articular cartilage against the progression of OA.

## INTRODUCTION

Statin (HMG-CoA reductase inhibitors) are widely used therapeutically to reduce morbidity and mortality in patients with hyperlipidemic cardiovascular disease [1,2]. More recently, it has been recognized that the benefit of statin therapy is not only based on lipid reduction, but also involves direct vascular effects [2,3]. In addition, anti-inflammatory activity and prevention of thrombosis are major determinants of the statin's benefit in atherosclerotic vascular disease [4-6]. Recent reports have revealed that statin modify apoptosis in vascular endothelial cells, resulting in altered vascular function [7]. It has been demonstrated that the effect of statin on matrix metalloproteinases (MMPs) is dependent on the inhibition of mevalonate synthesis, resulting in production of the mevalonate isoprenoid derivatives, farnesyl pyrophosphate and geranylgeranyl pyrophosphate [8-10]. These properties indicate a potential to modify inflammatory disease states such as rheumatoid arthritis (RA).

Leung et al. reported that simvastatin markedly inhibited not only developing but also established collagen-induced arthritis in a mouse model [11,12]. Another report also suggested that statin, by blocking HMG-CoA-reductase, inhibited production of MMPs by cultured chondrocytes [13]. Moreover, it has been shown that mevastatin increases the mRNA levels of bone morphogenetic protein-2, aggrecan and collagen type II as well as increasing proteoglycan synthesis in rat chondrocytes [14]. These findings suggest a possible additional mechanism for statin in counteracting osteoarthritis (OA) involving cartilage degeneration. However, further studies are needed to clarify the exact effect of statin on the pathogenesis of OA.

Based on these properties of statin, we hypothesized that they may have the potential to prevent degeneration of articular cartilage. We focused on the involvement of chondrocyte aging in articular cartilage degeneration [15]. Recently, the idea that chondrocyte aging is closely associated with the progression of cartilage degeneration has been promulgated [16-19]. Several reports of age-related changes in articular cartilage are consistent with the phenotypes of chondrocyte senescence, such as telomere shortening and changes in cell morphology, cellular viability and matrix protein production, suggesting that chondrocyte aging occurs with donor aging [17-19]. Here, we demonstrate that statin can protect against catabolic stress-induced disability and cellular aging in human articular chondrocytes *in vitro* and has potential as a protective agent against degeneration of articular cartilage during the progression of osteoarthritis (OA) *in vivo*.

## RESULTS

### Statin inhibits IL-1β-induced aging of articular chondrocytes

**Figure 1.**
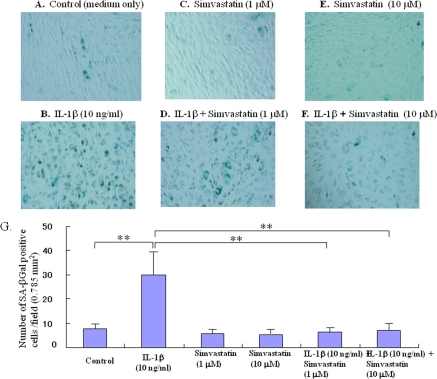
Effect of statn on chondrocyte aging *in vitro.* Chondrocyte senescence was measured by the senescence-associated β-galactosidase (SA-β-Gal) activity assay. The chondrocytes were cultured with IL-1β (10 ng/ml) in the presence or absence of statin (1.0, 10.0 μM) for 5 days. (**A-F**) Representative images of SA-β-Gal staining, (**A**) control (medium only), (**B**) IL-1β-treated group, (**C**) statin (1.0 μM)-treated group, (**D**) IL-1β + sratin (1.0 μM)-treated group, (**E**) statin (10.0 μM)-treated group, (**F**) IL-1β + sratin (10.0 μM)-treated group. As shown in the representative images, whereas IL-1β stimulated the expression of SA-β-Gal in chondrocytes, treatment with statin markedly inhibited the expression of SA-β-Gal even in the presence of IL-1β stimulation. (**G**) Treatment with statin significantly inhibited the IL-1β-enhanced SA-β-Gal activity in the chondrocytes.

The phenotype of IL-1β-induced cell senescence was characterized by an increase in SA-β-Gal activity. IL-1β enhanced cellular SA-β-Gal activity in comparison with controls (Figure 1A, B, G, p< 0.01). In contrast, treatment with statin (1.0 or 10.0 μM, Figure 1C-F) reduced the IL-1β-induced expression of this senescence marker in chondrocytes (Figure 1G, p< 0.01 compared to the IL-1β-stimulated group).

**Table 1.** Inhibitory effect of statin to chondrocyte life-span

Group	*In vitro* life-span (remaining replicative capacity) Populating doubling
Control	18,5 ± 3,4
IL-β	12,6 ± 3,6 ^a^
Simvastatin 1Mμ	19,4 ± 3,3
Simvastatin 10 Mμ	16,8 ± 5,0
IL-1 (10 ng/ml) + Simvastatin 1Mμ	20.3 ± 4.2 ^b^
IL-1 (10 ng/ml) + Simvastatin 1Mμ	17.8 ± 3,0 ^c^

a p < 0.01, compared to control

b and c p < 0.01 compared to IL-1β-stimulated group

### Statin maintains the life span of chondrocytes

The mean life-span of chondrocytes in the absence of IL-1β-stress was 18.5 ± 3.4 PD (n = 4), whereas the mean life-span of IL-1β-stressed chondrocytes was 12.6 ± 3.6 PD (n = 4, p < 0.05 compared to control). Treatment with statin (1.0 or 10.0 μM), however, prevented the IL-1β-induced shortening of life-span (Table 1).

### Statin inhibits the catabolic factor-induced production of matrix degrading enzymes

**Figure 2.**
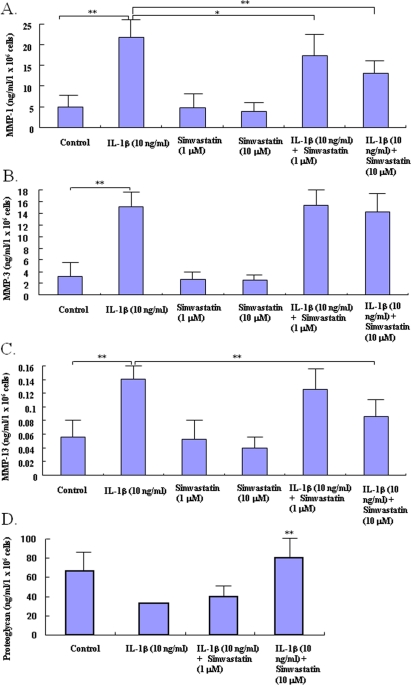
Effects of statin on the production of matrix degrading enzymes and proteo-glycan in chondrocytes. Chondrocytes were incubated with catabolic factor, IL-1β (10 ng/ml) in the presence or absence of statin (1.0, 10.0 μM). After 48 hour-incubation, the concentrations of matrix degrading enzymes and proteoglycan were analyzed by ELISA (A: MMP1, B: MMP-3, C: MMP-13, D: proteoglycan). IL-1β stimulated the production of MMP-1, -3, and -13 by chondrocytes in control cultures (* p< 0.05 compared to each control). Statin at 10.0 μM significantly decreased the IL-1β- induced excess production of MMP-1 and -13, but not MMP-3, from chondrocytes (** p< 0.01, compared to IL-1β-stimulated group). IL-1β showed a tendency to decrease the production of proteoglycan by chondrocytes. Statin at 10.0 μM cancelled the IL-1βinduced decreases of proteoglycan production from chondrocytes in both culture conditions (** p< 0.0, compared to IL-1β-stimulated group). Data from four independent experiments were analyzed.

IL-1β enhanced production of MMP-1, -3 and -13 in cultured chondrocytes (Figure 2A, B, C). Statin at 10.0 μM significantly inhibited the IL-1β-induced increase in production of MMP-1 and -13 (Figure 2A, C), but not MMP-3 (Figure 2B), in chondrocytes.

### Proteoglycan production from cultured chondrocytes

To test whether statin-mediated changes affect the potential of chondrocytes to produce matrix protein, we determined the amount of proteoglycan produced by the statin-treated chondrocytes. Whereas treatment with IL-1β caused a slight decrease in proteoglycan production by chondrocytes, treatment with statin at 1.0 μM showed a tendency to maintain proteoglycan production even in the presence of IL-1β (Figure 2D). Treatment with statin at 10.0 μM significantly increased the production of proteoglycan even in the IL-1β-stressed chondrocytes (P< 0.05 compared to control).

### Treatment with statin protects against the development of cartilage degeneration in OA model mice

**Figure 3.**
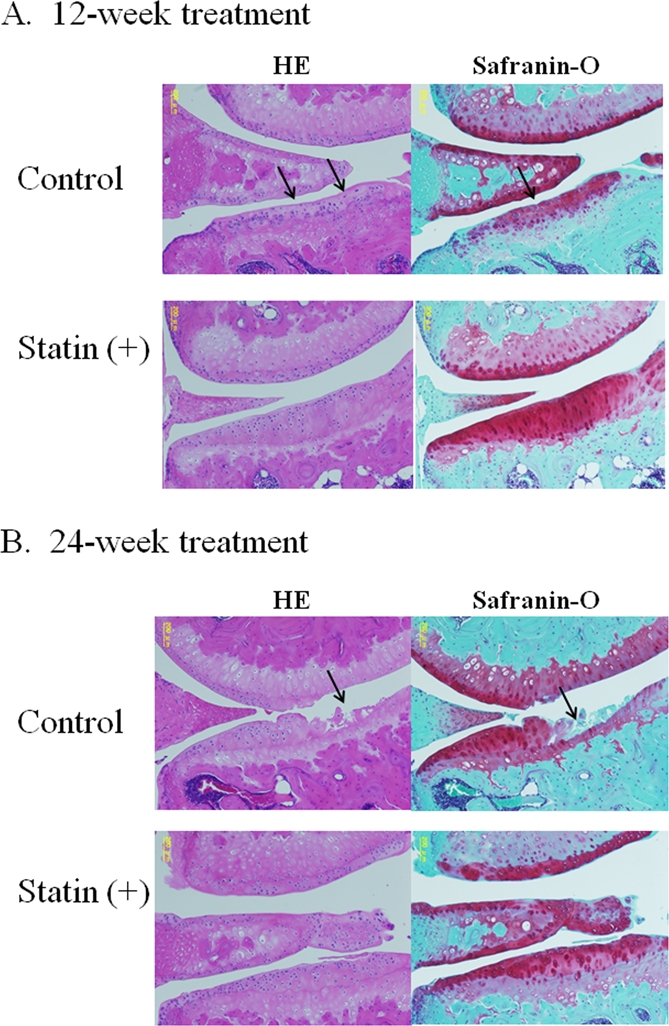
Representative images of cartilage degeneration in OA model mouse. (**A**) At 12 weeks, the cartilage in the control group showed a progression of following cartilage degeneration: chondrocyte clustering, abnormal deposition of chondrocytes, and a decreased cell density with cartilage thinning. The cartilage in the statin-treated mice showed less severe in contrast to the control group. (**B**) After 24-week treatment, most of the control cartilage showed decrease in thickness, marked loss of proteoglycan, chondrocyte clustering, cell death and severe degeneration of cartilage structure, whereas treatment with statin markedly inhibited the degeneration of articular cartilage in the OA mouse model.

**Figure 4.**
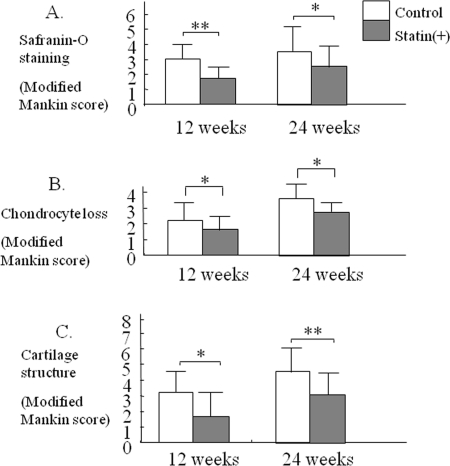
Therapeutic effect statin in OA mouse model. Each cartilage sample was evaluated histologically for the degree of degeneration, loss of Safranin-O green staining, chondrocyte loss, cartilage structure, according to a modified Mankin grading system (Table 2). At 12 and 24 weeks, severe damage in articular cartilage was observed in each control joint. Treatment with statin induced a significant reduction of cartilage degeneration in comparison with control (* p< 0.05, ** p< 0.01).

In the control joints, after 12 and 24 weeks the following changes were observed: An extension of the area of surface fibrillation, chondrocyte clustering, abnormal distribution of chondrocytes, reduced proteoglycan staining and decreased cell density with cartilage thinning (Figure 3A, B). After 24 weeks of treatment, most of the control cartilage showed a decrease in thickness, a marked loss of proteoglycan, chondrocyte clustering and cell death (Figure 3B). As shown in the representative images shown in Figure 4 A and B, treatment with statin markedly inhibited the degeneration of articular cartilage in the OA mouse model.

At 12 weeks, the degree of cartilage degeneration tended to be more severe in the control group than in the statin treated group. At 24 weeks, mild cartilage damage was observed in the statin treated group, whereas more severe damage was observed in the control group (Figure 4, * p< 0.05, ** p< 0.01 compared to the control group).

## DISCUSSION

The studies reported here sought to ascertain whether statin can function as a protective agent against catabolic stress-induced degeneration of articular cartilage in an OA model *in vitro* and *in vivo*. Our results indicate that statin inhibits the following catabolic responses: production of matrix degrading enzymes (MMP-1 and -13), down-regulation of production of matrix proteins and cellular senescence in chondrocytes stressed by the OA-inducing factor, IL- 1β. In addition, we found that statin significantly reduced articular cartilage degeneration in an OA mouse model, an experimental model which spontaneously develops an osteoarthritic process.

In the present study, we demonstrate that statin has the ability to inhibit senescence of articular chondrocytes stressed by the catabolic factor, IL-1β. Our results indicate that treatment with statin prevents acquisition of the phenotype associated with cellular senescence which is accelerated by IL-1b treatment. Treatment with statin decreased the enhancement of SA-β-Gal activity induced by IL-1β.In addition, statin inhibited the IL-1β-induced shortening of the lifespan of cultured chondrocytes.

Recent studies on human articular chondrocytes from donors ranging in age from 1 to 87 years showed that senescence-associated beta-galactosidase (SA-β-Gal) activity increased with age, whereas both mitotic activity and mean telomere length declined [17]. Previous reports indicated that chondrocyte senescence occurs throughout life *in vivo* [18,19]. This indirect evidence supports the hypothesis that age-related changes in articular cartilage increase the vulnerability of the tissue to degeneration and that the association between OA and aging is due, at least in part, to chondrocyte senescence. Furthermore, increased SA-β-Gal activity was observed in damaged OA cartilage, and mean telomere length was lower in cells near the lesion compared to distal sites in the same joint [19,20]. These findings suggest that chondrocyte senescence, at least in part, participates in the pathogenesis of OA. However, the exact mechanism of chondrocyte senescence and its implications for the pathogenesis of OA still remain unclear.

Cellular senescence is classified into 2 types, intrinsic telomere-dependent replicative senescence and extrinsic telomere-independent senescence. There is a general consensus that extrinsic senescence is induced by several types of stress, such as oxidative stress or pro-inflammatory cytokines [21-23]. Numerous catabolic stresses involving mechanical loading, cytokines, and oxidative stress are involved in the pathophysiology of OA. Our previous study revealed that catabolic factors may result in extrinsic stress-induced senescence of articular chondrocytes [24]. In the current study, our data on the inhibitory effects of statin on chondrocyte senescence (stress-induced extrinsic senescence) may be important in devising new approaches to the prevention and treatment of OA, although further studies are needed to clarify the mechanism responsible for the inhibitory effect of statin on chondrocyte aging. Statin is known to have anti-oxidative and anti-inflammatory potential [2,3,11,12]. The inhibitory effect of statin on IL-1β-induced chondrocyte senescence may be based on its anti-inflammatory or anti-oxidative potential. Data from other studies suggest that the anti-oxidative and anti-inflammatory properties of statin may vary. Thus, further studies could be needed to analyze whether these effects of statin represent a class effect or are specific for particular statin.

In our previous study, we demonstrated that catobolic factors acting on articular cartilage, such as proinflammatory cytokines and oxidative stress, induced senescent cellular phenotypes in chondrocytes: Changes in cell morphology, shortening of cellular replicative lifespan, telomere shorting and cell cycle arrest [15] suggesting that OA-related catabolic factors accelerate chondrocyte senescence and the related degeneration of articular cartilage. However, it still remains unclear why catabolic factors induce chondrocyte senescence. Recent studies indicate that activation of mTOR (target of rapamycin) is closely involved in cellular senescence [25-37]. Demidenko et al. clearly demonstrated that growth stimulation coupled with cell cycle arrest leads to senescence, whereas quiescence (a condition with inactive TOR) prevents senescence [26,27]. They also demonstrated that an inhibitor of mTOR, rapamycin, decelerates cellular senescence [25]. Dumont et al. reported that proliferation of T cells was stimulated by IL-1 and that rapamycin significantly decreased the IL-1-stimulated proliferation of T cells, suggesting that IL-1 stimulates mTOR pathway [28]. This suggests that IL-1 accelerates the cellular senescence. The activation of mTOR may thus be implicated in chondrocyte senescence in OA. Although further studies are needed to clarify the exact mechanism of catabolic stress-induced chondrocyte senescence, the activation of mTOR may be implicated in the chondrocyte senescence in OA.

In conclusion, our *in vitro* and *in vivo* findings suggest that statin may have the potential to prevent catabolic stress-induced downregulation of chondrocyte activity and accelerated cellular aging in articular cartilage. Statin may thus have a therapeutic effect on articular cartilage degeneration.

## MATERIALS AND METHODS

### Isolation and culture of chondrocytes

Full-thickness human articular cartilage samples were collected from the joints of 4 individuals with OA (70, 72, 76, 77 years old) who underwent arthroplastic knee surgery. Informed consent was obtained in accordance with the local ethical commission requirements. The harvested cartilage samples were chopped up and digested overnight in Dulbecco's modified Eagle's medium (DMEM), containing 0.5 mg/ml of pronase E and 0.5 mg/ml of type II collagenase at 37°C. The resulting single cell suspension was filtered through a 70-μm mesh nylon strainer, and the cells were incubated at 37°C in a 5% CO_2_ humidified atmosphere in DMEM containing 10% FCS, 2 mM L-glutamine, 100 units/ml penicillin and 100 μg/ml streptomycin. To avoid loss of the chondrocyte phenotype with successive passages, we used the cultured chondrocytes at passage 1 - 3.

### Senescence-associated β-galactosidase (SA-β-Gal) activity

When cells reached subconfluence, the monolayers were subjected to enzymatic dissociation with trypsin and seeded onto new dishes. In the presence or absence of titrated concentrations of statin (1.0 or 10.0 μM, Kaken Pharm. Co. Ltd., Tokyo, Japan), the chondrocytes were stimulated with 10 ng/ml interleukin (IL)-1β for 5 days.

To investigate senescence, the SA-β-gal activity in cultured chondrocytes was detected as previously described [29], using the Senescence Detection Kit (BioVision, Mountain View, CA) according to the manufacturer's recommendations. Cells were photographed under reflected light using a VH-8000 digital high fidelity microscope (Keyence, Osaka, Japan). The number of SA-β-gal positive cells/field was counted in 10 fields/slide and the mean number of positive cells/field (x 200) was calculated.

### Cellular lifespan of chondrocytes

The cells were subcultured every 4 days in the presence or absence of statin (1.0 or 10.0 μM). At each subculture, the total number of cells was determined by counting in a haemocytometer, and cells were transferred to new dishes at a ratio of 1:3. The non-adherent cells were counted 6 h post-seeding, and their number subtracted from the number of seeded cells. The increase in cumulative population doublings at each subculture was calculated based on the number of cells attached and the cell yield at the time of the next subcultivation. The end of the replicative lifespan was defined by failure of the population to double after 4 weeks in mass culture. The *in vitro* life-span (remaining replicative capacity) was expressed as population doublings (PD) up to cellular senescence. Population doublings were calculated using the following equation: PD = Log_10_ (N/N_0_) ×3.33, where N is the number of cells at the end and N_0_ is the number of cells at the beginning of the experiment [29,30]. To maintain the phenotypic characteristics of the chondrocytes during serial passage for determination of life-span, we used CGM^TM^ BulletKit^®^(chondrocyte growth medium) instead of DMEM.

**Table 2.** Modified Mankin score (criteria for histological evaluation).

Safranin O-fast green staining 0 = uniform staining throughout articular cartilage 1 = loss of staining in the superficial zone for less than one-half of the length of the plateau 2 = loss of staining in the superficial zone for one-half or more of the length of the plateau 3 = loss of staining in the superficial and middle zones for less than one-half of the length of the plateau 4 = loss of staining in the superficial and middle zones for one-half or more of the length of the plateau 5 = loss of staining in all 3 zones for less than one-half of the length of the plateau 6 = loss of staining in all 3 zones for one-half or more of the length of the plateau
Chondrocyte loss 0 = no decrease in cells 1 = minimal decrease in cells 2 = moderate decrease in cells 3 = marked decrease in cells 4 = very extensive decrease in cells
Structure 0 = normal 1 = surface irregularities 2 = 1-3 superficial clefts 3 = >3 superficial clefts 4 = 1-3 clefts extending into the middle zone 5 = >3 clefts extending into the middle zone 6 = 1-3clefts extending into the deep zone 7 = >3 clefts extending into the deep zone 8 = clefts extending to calcified cartilage

### Measurement of anabolic and catabolic activity of chondrocytes

To measure the catabolic activity of chondrocytes, levels of MMP-1, -3 and -13 produced by chondrocytes were measured using an ELISA kit (MMP-1: Amersham Biosciences, Buckinghamshire, UK; MMP-3: R&D Systems Inc., Minneapolis, MN; MMP-13: Amersham Biosciences).

To examine the effect of statin on anabolic activity in chondrocytes, the levels of proteoglycan produced by chondrocytes in culture were measured using an enzyme-linked immunosorbent assay (ELISA) kit in accordance with the manufacturer's protocol (BioSource Europes S.A., Nivelles, Belgium).

### Animals and administration protocols

The study was performed on STR/OrtCrlj mice, an experimental model strain which spontaneously develops an osteoarthritic process. STR/OrtCrlj mice (male, 12 weeks of age) were purchased from Charles River Japan (Yokohama, Japan). The mice were maintained in a temperature (23-25 °C)-, humidity (40-60%)-, and light-controlled environment with free access to an MF diet(Japan SLC Co. Ltd., Shizuoka, Japan) and water. They were acclimatized for at least 1 week before the start of the study. Twenty-four animals were divided into 2 groups (statin-fed or control). The statin-fedgroup received 40 mg/kg/day of statin by daily gavage for 24 weeks. The control group were given saline by daily gavage for 24 weeks. The care and use ofthe experimental animals in this study followed “The EthicalGuidelines of Animal Care, Handling and Termination” prepared by the National Institute of Health Sciences of Japan. The mice were euthanized after 12 or 24 weeks of treatment and both knee joints were harvested. Bone and cartilage tissue blocks were prepared for histological analysis.

### Articular cartilage lesion score

Each cartilage sample was evaluated histologically for the degree of degeneration according to a modified Mankin grading system [31]. Articular cartilage samples together with subchondral bone were fixed for 2 days in 4% paraformaldehyde solution and then decalcified in 4% paraformaldehyde containing 0.85% sodium chloride and 10% acetic acid. Tissues were dehydrated through a series of ethanol solutions and infiltrated with xylene before being embedded in paraffin and cut into 6 μm sections. Sections were deparaffinized through sequential immersion in xylene and a graded series of ethanol solutions in accordance with conventional procedures. The sections were stained with Safranin O-fast green or hematoxylineosin. The mean damage score from the 6 - 8 samples from each animal was used to determine the mean ± standard deviation for each group. Three independent observers assessed cartilage damage in a blinded manner.

### Statistical analysis

Results are expressed as means ± standard deviations. Data were analyzed using a nonparametric statistical method. A *P* value < 0.05 was considered statistically significant.

Student's *t*-test was used to assess the differences between statin-treated and control groups with respect to histologic parameters (Safranin O-fast green staining, structure, chondrocyte loss; Table 2) of the tibial plateau in the isolated knee joints. When a statistically significant difference between the 2 groups was detected, analysis of variance (ANOVA) was used to adjust for the confounding effects of animals and observers.
